# Ad5-nCoV Vaccination Could Induce HLA-E Restricted CD8^+^ T Cell Responses Specific for Epitopes on Severe Acute Respiratory Syndrome Coronavirus 2 Spike Protein

**DOI:** 10.3390/v16010052

**Published:** 2023-12-28

**Authors:** Yuling Wang, Lu Yang, Kang Tang, Yusi Zhang, Chunmei Zhang, Yun Zhang, Boquan Jin, Yuan Zhang, Ran Zhuang, Ying Ma

**Affiliations:** Department of Immunology, Air Force Medical University, Xi’an 710032, China; wangyuling@fmmu.edu.cn (Y.W.); yangluer@fmmu.edu.cn (L.Y.); immukang@fmmu.edu.cn (K.T.); immuzys@fmmu.edu.cn (Y.Z.); hzcm1981@126.com (C.Z.); immuzy@fmmu.edu.cn (Y.Z.); immu_jin@fmmu.edu.cn (B.J.); zhangyuan@fmmu.edu.cn (Y.Z.)

**Keywords:** severe acute respiratory syndrome coronavirus 2, Ad5-nCoV, vaccination, HLA-E, CD8^+^ T cell response, epitope

## Abstract

We evaluated cellular immune responses induced by severe acute respiratory syndrome coronavirus 2 (SARS-CoV-2) vaccines in an immunized population based on HLA-E-restricted CD8^+^ T cell epitope identification. HLA-E-restricted SARS-CoV-2 CD8^+^ T cell nonamer peptides were predicted with software. An HLA-E-transfected K562 cell binding assay was used to screen for high-affinity peptides. IFN-γ enzyme-linked immunospot assays were used to identify HLA-E-restricted epitopes. An HLA-E/epitope tetramer was employed to detect the frequencies of epitope-specific CD8^+^ T cells. Four CD8^+^ T cell epitopes on the spike protein of SARS-CoV-2 restricted by both HLA-E*0101 and E*0103 were identified. HLA-E-restricted epitope-specific IFN-γ-secreting CD8^+^ T cell responses could be detected in individuals vaccinated with SARS-CoV-2 vaccines. Importantly, the frequencies of epitope-specific CD8^+^ T cells in Ad5-nCoV vaccinated individuals were higher than in individuals vaccinated with recombinant protein or inactivated vaccines. Moreover, the frequencies of epitope-specific CD8^+^ T cells could be maintained for at least 120 days after only one dose of Ad5-nCoV vaccine, while the frequencies of epitope-specific CD8^+^ T cells decreased in individuals after two doses of Ad5-nCoV vaccine. These findings may contribute to a more comprehensive evaluation of the protective effects of vaccines for SARS-CoV-2; meanwhile, they may provide information to characterize HLA-E-restricted CD8^+^ T cell immunity against SARS-CoV-2 infection.

## 1. Introduction

The acute infectious coronavirus disease 2019 (COVID-19), caused by severe acute respiratory syndrome coronavirus 2 (SARS-CoV-2) infection, is characterized by fever and upper respiratory symptoms, and even multiple organ failure, such as acute respiratory distress syndrome [1]. COVID-19 began to appear in December 2019 and posed a huge threat to global public health and human life. The World Health Organization (WHO) declared that COVID-19 had become a global pandemic in March 2020. COVID-19 spread across nearly every country. Up to now, COVID-19 has accumulated more than 661 million confirmed cases and more than 6.7 million deaths worldwide.

Vaccination is the most effective way to prevent SARS-CoV-2 infection. At present, many types of vaccines against SARS-CoV-2 have been widely used in the world and have achieved certain preventive effects. Among them, a recombinant SARS-CoV-2 vaccine with an adenovirus type 5 vector, which was developed and produced in China (Ad5-nCoV, CanSinoBIO, Tianjin, China), has been proven to induce a high level of both humoral and cellular immune responses [2,3,4]. Importantly, Ad5-nCoV has been widely applied in the population and was approved by WHO as a COVID-19 vaccine on the “Emergency Use List” in May 2022 [5,6,7].

SARS-CoV-2 is a single positive-stranded, enveloped RNA virus that can infect many animal species and humans. The genome contains six open reading frames (ORFs), which encode non-structural proteins such as RNA-dependent RNA polymerase (RdRp), and four structure proteins, namely spike protein (S), envelope protein (E), membrane protein (M), and nucleocapsid proteins (N) [8]. The S protein can induce a high level of specific antibodies. However, antibody responses against SARS-CoV-2 cannot be well-maintained for a long time [9].

It has been reported that SARS-CoV-2-specific CD8^+^ T cell responses in the peripheral blood of COVID-19 patients are closely related to remission of the disease [10,11]. In fact, SARS-CoV-2 specific CD8^+^ T cell responses can be detected in the acute phase of COVID-19 within 7 days after the onset of symptoms, reaching their highest level at 14 days [12]. Studies in SARS-CoV-2-infected animal models have found that although neutralizing antibodies can protect against viral attack, CD8^+^ T cell responses can provide more important clinical protection when antibody levels are low or reduced [13]. CD8^+^ T cell epitopes of SARS-CoV-2 restricted by classical HLA-I molecules, such as HLA-A1/ORF1a1637, HLA-A2/S269, HLA-A3/N361, HLA-A24/S1208, HLA-B7/N105, and HLA-B40/N322, were used to further study the effects and mechanisms of specific CD8^+^ T cell responses in SARS-CoV-2 infection [14,15]. Among them, the NP105-113 epitope restricted by HLA-B7 has been proven to induce a specific CD8^+^ T cell response with strong antiviral activity [16]. Although most studies focused on classical HLA-I restricted CD8^+^ T cell epitopes and specific responses, non-classical HLA-I molecule HLA-E restricted CD8^+^ T cells also play an important role in immune protection and regulation in many viral infectious diseases [17,18,19]. Notably, HLA-E is a ubiquitous HLA locus with only two different alleles, HLA-E*0101 and HLA-E*0103, both of which have a combined frequency of over 99% in the global population. These two molecules just differ in position 107 of the mature HLA-E protein (arginine for HLA-E*0101 and glycine for HLA-E*0103) [20]. It has been proposed to use HLA-E restricted CD8^+^ T cells to treat severe COVID-19 patients in the early stage of SARS-CoV-2 infection [21]. Therefore, it is necessary to identify CD8^+^ T cell epitopes that can overcome the polymorphism of HLA molecules. Moreover, the detection of SARS-CoV-2 epitope-specific CD8^+^ T cell responses may be important for the evaluation of the protective effects induced by SARS-CoV-2 vaccines.

In this study, specific CD8^+^ T cell responses in different vaccine-inoculated populations, especially in Ad5-nCoV vaccinated subjects, were detected based on the identification of four epitopes of the S protein of SARS-CoV-2 restricted by both HLA-E*0101 and E*0103. These findings may provide crucial information to evaluate the effects of COVID-19 vaccines in immunized populations according to HLA-E-restricted SARS-CoV-2 specific CD8^+^ T cell responses.

## 2. Materials and Methods

### 2.1. Study Cohort

A total of 56 samples from 43 individuals vaccinated with SARS-CoV-2 vaccines were enrolled in this study. The ages of the vaccinated subjects ranged between 21 and 32 years. Any vaccinated donors who were suffering from infectious diseases or autoimmune diseases, or experiencing any inflammation or infection within a week before sampling, were excluded from this study. All participants had never been infected with SARS-CoV-2 before participating in the experiment and provided ongoing nucleic acid test certificates. By performing indirect ELISA assays on the serum obtained from the collected blood samples, which targets the S protein (40591-V08H, SinoBiological, Beijing, China) and N protein (B234601, Biointron, Shanghai, China) of SARS-CoV-2, it was ascertained that the subjects had not encountered any exposure to the virus. The antibodies used in the indirect ELISA were all prepared by our research group [22]. Subjects were divided into three groups according to the different types of vaccine they received, including the inactivated vaccine group (CoronaVac, Sinovac Biotech, Beijing, China), recombinant protein subunit vaccine group (ZF2001, Zhifei Biological, Chongqing, China), and the adenovirus-vectored vaccine group (Ad5-nCoV, CanSino Biologics, Tianjin, China). The subjects who received the adenovirus-vectored vaccine (Ad5-nCoV, CanSino Biologics, Tianjin, China) were administered their initial dose on 11 March 2021, followed by a subsequent dose on 3 December 2021. The interval between these administrations spanned a duration of 267 days. The basic information of the enrolled volunteers is summarized in Table 1.

### 2.2. Sample Collection

Peripheral venous blood samples of SARS-CoV-2 vaccinated individuals were drawn into pyrogen-free blood collection tubes with citrate as an anticoagulant. Peripheral blood mononuclear cells (PBMCs) were isolated using standard Ficoll-Hypaque (Sigma-Aldrich, St. Louis, MO, USA) density gradient centrifugation.

### 2.3. Epitopes Prediction

The Immune Epitope Database and Analysis Resource (IEDB), NetMHC4.0 Server, and NetMHCpan 4.1 Server were used to predict the binding of peptides to MHC molecules of known sequences using artificial neural networks (ANNs). Specifically, a nine-mer peptide as the peptide length option was selected. HLA-E*0101 or HLA-E*0103-restricted SARS-CoV-2 nine-mer peptide epitopes were predicted by inputting 1273 amino acids of the S protein sequence, 75 amino acids of the E protein sequence, 222 amino acids of the M protein sequence, and 419 amino acids of the N protein sequence of the SARS-CoV-2 prototype strain Wuhan-Hu-1 (GenBank accession number: NC_045512.2) into the databases.

Only the HLA-E*0101 allele option could be selected in the NetMHC4.0 Server database, while both the HLA-E*0101 and E*0103 alleles could be selected in the NetMHCpan 4.1 Server database under the loci or species option with HLA-E. Both methods specified if a sequence is a strong MHC binder (SB) or a weak MHC binder (WB) based on a %Rank score. By default, %Rank < 0.5% and %Rank < 2% thresholds are considered for detecting SB and WB for an HLA-E molecule.

### 2.4. Peptides Synthesis

The predicted SARS-CoV-2 nine-mer epitopes were synthesized to at least 90% purity as assessed by high-performance liquid chromatography and mass spectrometry (ChinaPeptides, Shanghai, China). Peptides from the HLA-Cw*03_3-11_ or HLA-A*02_3-11_ leading sequences VMAPRTLIL or VMAPRTLVL, which have been confirmed to be the HLA-E-restricted epitopes, were synthesized and used as positive controls. The peptide melanoma antigen-encoding gene (MAGE-1) with sequence EADPTGHSY, which cannot bind to HLA-E molecules, was synthesized and used as a negative control. All of the peptides were stored at 1 mM concentration at −70 °C and repeated freeze-thawing was avoided.

### 2.5. K562 Cell Lines Transfected with HLA-E Molecule

An immortalized chronic myelogenous leukemia cell line K562 with MHC molecule expression deficiency was obtained from ATCC (CCL-243). We stably transfected the K562 cell line with single allele HLA-E*0103 or HLA-E*0101 to generate mono-allelic cell lines with lentivirus transfection methods. Stably transfected cells showed strong expression (>95%) of HLA-E*0103 or HLA-E*0101 according to flow cytometry (FCM) analysis.

### 2.6. K562/HLA-E Cell Binding Assay

The stable transformed K562/HLA-E*0103 or K562/HLA-E*0101 cells were respectively incubated with 10 μM of each indicated peptide and 1 μM human β2-microglobulin (β2m, Sigma, St. Louis, MO, USA) in serum-free RPMI 1640 medium for 16 h at 26 °C with 5% CO_2_, while K562/HLA-E cells with no peptide incubation under the same culture conditions were used as a blank control. The cells binding with peptides were further incubated at 37 °C for 2 h for thermal stability. We detected the expression of HLA-E molecules on the surface of K562/HLA-E cells by staining with PE-labeled anti-HLA-E monoclonal antibody (mAb) (3D12; BioLegend, San Diego, CA, USA), using an ACEA NovoExpress system (Agilent Technologies, Palo Alto, CA, USA). The results are presented as the fluorescence index (FI). FI ≥ 1 represents a high-affinity peptide, indicating that the stable combination of the peptide with HLA-E molecules on the surface of K562/HLA-E cells could increase the mean fluorescence of the HLA-E molecules by at least one-fold.

### 2.7. Isolation of CD8^+^ T Cells with Magnetic Bead Kits

The CD8^+^ T cells from PBMCs were isolated by magnetic streptavidin nanobeads from Biolegend MojoSort^TM^ Isolation Kits (Cat:480012). PBMCs were incubated with a biotin–antibody cocktail followed by incubation with the magnetic streptavidin nanobeads. The targeted cells were depleted by the magnetically labeled fraction using a magnetic separator, while the untouched CD3^+^CD8^+^ cells were collected. The aforementioned operations were executed in strict accordance with the provided instructions.

### 2.8. K562/HLA-E Cells Pre-Incubation with Peptides

Before incubation with peptides, K562/HLA-E*0103 cells were incubated at 26 °C for 24 h to adapt to the conditions for antigen presentation in advance. The K562 cells were then incubated with the peptides (1 μL/10^6^ cells) at 26 °C for 19 h until the next assay.

### 2.9. Ex Vivo IFN-γ Enzyme-Linked Immunospot Assay (ELISpot)

A human IFN-γ ELISpot kit (DAKEWE Biotech Company, Shenzhen, China) was used to detect the capacity of IFN-γ production by CD8^+^ T cells stimulated with specific antigenic peptides presented by K562/HLA-E cells as previously described. The spots representing peptide-specific IFN-γ-producing CD8^+^ T cells were counted using an automatic ELISpot reader (Cellular Technology Limited, Cleveland, OH, USA). The adjusted spot-forming cells (SFCs) after subtracting the negative values are expressed as SFCs/10^6^ cells.

### 2.10. Peptide/HLA-E*0103 Tetramer Staining

Two peptides with high binding affinity (S17 and S19) were selected for the construction of a peptide/HLA-E tetramer labeled with PE by Epigen Biotech (Nantong, China). The PBMCs of the SARS-CoV-2 vaccinated individuals were stained with each PE-labeled peptide/HLA-E tetramer for 10 min and subsequently stained with FITC-labeled anti-human CD8 mAb (clone SK1, Biolegend, San Diego, CA, USA) and APC-labeled anti-human CD3 mAb (clone OKT3, Biolegend, San Diego, CA, USA). Approximately 300,000 cells gated on CD3^+^ cells were captured. The gate for CD8^+^ tetramer^+^ T cells was set up by matching the non-tetramer staining isotype control (BioLegend). Compensation controls were checked regularly to avoid false-positive results and individually determined for each experimental setup.

### 2.11. Statistical Analysis

Statistical analysis was performed using GraphPad Prism software, version 6 (GraphPad; La Jolla, CA, USA). The Mann–Whitney *U* test was used for parameter comparisons between two groups. The frequencies of epitope-specific CD8^+^ T cells are presented as the mean with 95% confidence interval (95% CI) or median with range. *p*-values (two-tailed) below 0.05 (*p* ≤ 0.05) were considered to be statistically significant.

## 3. Results

### 3.1. Thirty-Six HLA-E-Restricted Peptides on SARS-CoV-2 Were Predicted and Synthesized

HLA-E*0101 or HLA-E*0103-restricted peptides from the SARS-CoV-2 structure proteins S protein, E protein, M protein, and N protein were predicted. According to the binding affinity, 36 predicted nonapeptides on SARS-CoV-2 with strong binding or weak binding ability in either of the two databases were selected. A total of 36 SARS-CoV-2-derived HLA-E-restricted nonapeptides with detailed information, including 25 peptides on S protein, 1 peptide on E protein, 6 peptides on M protein, and 4 peptides on N protein are listed in Table S1.

### 3.2. Four Nonapeptides of SARS-CoV-2 Exhibited High Binding Affinity to Both HLA-E*0103 and HLA-E*0101 Molecules

**Figure 1:**
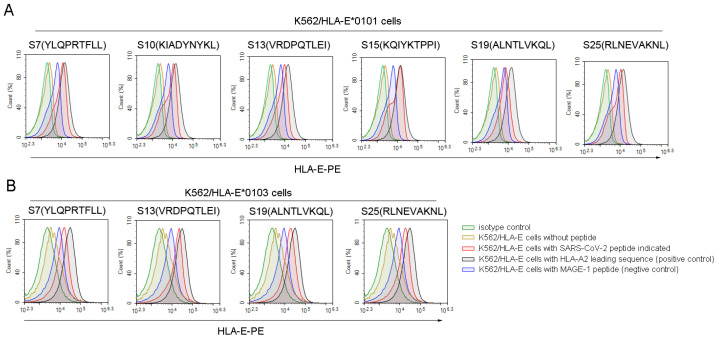
High binding affinity of SARS-CoV-2 nonapeptides to the HLA-E*0103 and HLA-E*0101 molecules. K562/HLA-E cells incubated with each peptide and β2-microglobulin were then stained with PE-labeled anti-HLA-E mAb and detected by flow cytometry. The red curves indicate K562/HLA-E cells incubated with each SARS-CoV-2 nonapeptide. The black curves indicate HLA-E molecule stabilization with HLA-E restricted HLA leading sequences, which served as positive controls. The yellow curves indicate cells incubated without peptide. The blue curves indicate cells incubated with MAGE-1 peptide, serving as negative controls. The green curves indicate isotype controls. (**A**) The overlay curves of K562/HLA-E*0101 cells incubated with each of the SARS-CoV-2 nonapeptides. (**B**) The overlay curves of K562/HLA-E*0103 cells incubated with each of the SARS-CoV-2 nonapeptides. MAGE-1, melanoma-associated antigen 1.

Then, we used the K562/HLA-E cell binding assay to further screen the high binding affinity SARS-CoV-2 nonapeptides to HLA-E*0103 or HLA-E*0101 molecules. Among the 36 predicted HLA-E-restricted SARS-CoV-2 peptides, 6 peptides, including S7, S10, S13, S15, S19, and S25, could increase the expression of HLA-E*0101 on the K562/HLA-E cell surface as characterized by a fluorescence index (FI) ≥ 1, indicating a high binding affinity to the HLA-E*0101 molecule (Figure 1A). Moreover, 4 of the 23 predicted peptides including S7, S13, S19, and S25 could also increase the HLA-E*0103 expression on the K562/HLA-E cell surface with FI ≥ 1, suggesting a high binding affinity to the HLA-A*0103 molecule as well (Figure 1B). Therefore, the four nonapeptides S7, S13, S19, and S25 of SARS-CoV-2 exhibited high binding affinity to both HLA-E*0103 and HLA-E*0101 molecules. However, there may be slight differences in the binding affinity among these peptides according to their FI values. Specifically, S19 showed higher binding affinity to the HLA-E*0103 molecule, while S7 and S15 exhibited higher binding affinity to the HLA-E*0101 molecule. Detailed information for the screened peptides with high binding affinity is summarized in Table S2.

### 3.3. HLA-E Restricted Epitopes of SARS-CoV-2 Were Identified by Inducing Specific IFN-γ-Producing CD8^+^ T Cell Responses

**Figure 2:**
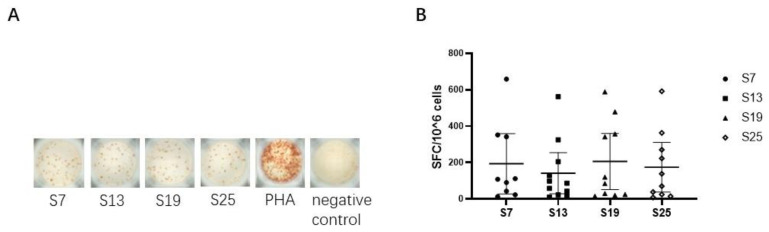
HLA-E-restricted epitopes of SARS-CoV-2 identified by inducing specific IFN-γ-producing CD8^+^ T cell responses in vaccinated individuals. The isolated CD8^+^ T cells were used as effector cells. K562/HLA-E cells pre-loaded with peptides were used as antigen-presenting cells (APCs). The ex vivo IFN-γ ELISpot assay was used to test the epitope-specific IFN-γ-producing CD8^+^ T cells in vaccinated individuals. (**A**) The representative IFN-γ-secretion spots of CD8^+^ T cells in vaccinated individual No. 32. CD8^+^ T cells were stimulated with K562/HLA-E cells pre-loaded with peptide or 10 μg/mL PHA in vitro. The notes under the wells indicate the SARS-CoV-2 peptides or the name of the stimulant. Cells stimulated with PHA served as the positive control, and cells with no stimulation were the negative control. (**B**) Comparison of the numbers of SFC among the four HLA-E restricted SARS-CoV-2 CD8^+^ T cell epitopes. The Kruskal–Wallis test was used to determine the significance of differences among the groups, and black lines represent the medians with the corresponding interquartile ranges. PHA, phytohemagglutinin. SFC, spot-forming cells.

We next assessed the capacity of each peptide to elicit epitope-specific CD8^+^ T cell responses by detecting the secretion of IFN-γ in vitro in SARS-CoV-2 vaccinated subjects. The results showed that four peptides (S7, S13, S19, and S25) could effectively elicit epitope-specific IFN-γ-secreting CD8^+^ T cell responses in the peripheral blood of vaccinated subjects (Figure 2A), indicating that the four SARS-CoV-2 peptides could be defined as HLA-E restricted CD8^+^ T cell epitopes. Specifically, the median (range) was 108 (13-685) SFC/10^6^ PBMCs for peptide S7, 87 (10-516) SFC/10^6^ PBMCs for peptide S13, 102 (15-588) SFC/10^6^ PBMCs for peptide S19, and 104 (8-591) SFC/10^6^ PBMCs for peptide S25. However, only 11 out of the 56 samples collected showed HLA-E-restricted epitope-specific CD8^+^ T cell responses. Moreover, the frequencies of IFN-γ-secreting CD8^+^ T cell responses showed no differences among the four HLA-E-restricted SARS-CoV-2 epitopes (Figure 2B).

### 3.4. The Frequencies of HLA-E-Restricted SARS-CoV-2 Epitope-Specific CD8^+^ T Cells Could Be Detected in the Peripheral Blood of Vaccinated Individuals

**Figure 3:**
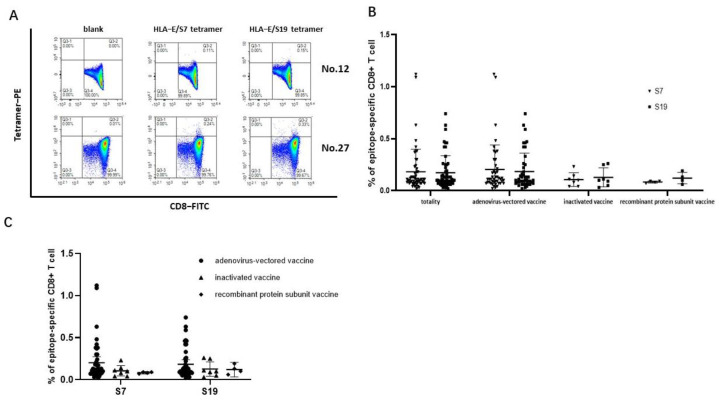
Frequencies of HLA−E-restricted SARS−CoV−2 epitope−specific CD8^+^ T cells in PBMCs of vaccinated individuals. The PBMCs from SARS-CoV-2 vaccinated individuals were stained with HLA-E*0103 tetramer preloaded with S7 or S19 peptide, respectively. HLA-E*0103/peptide tetramer+ cells gated from CD3^+^CD8^+^ T lymphocytes were considered epitope-specific CD8^+^ T cells. (**A**) Representative flow cytometric plots of SARS-CoV-2 epitope-specific CD8^+^ T cells of No. 12 and No. 27 vaccinated individuals. (**B**) Comparison of the frequencies of epitope-specific CD8^+^ T cells between the S7 and S19 epitopes in each vaccine group. (**C**) Comparison of the frequencies of epitope-specific CD8^+^ T cells among the three vaccinated groups for each epitope. The black lines represent the medians with the corresponding interquartile ranges. The Mann–Whitney *U* test was used for comparisons between two groups or two epitopes.

The SARS-CoV-2 epitopes S7 and S19 were selected to construct HLA-E/peptide tetramers to accurately detect the frequencies of epitope-specific CD8^+^ T cells in the peripheral blood of SARS-CoV-2 vaccinated subjects. The frequencies of both epitope S7 and epitope S19-specific CD8^+^ T cells could be detected (Figure 3A). The frequency of epitope S7-specific CD8^+^ T cells in 56 samples ranged from 0.020% to 1.120% (median values: 0.11%, 95% CI: 0.1234%–0.2398%), while the frequency of epitope S19-specific CD8^+^ T cells ranged from 0.020% to 0.74% (median value: 0.10%, 95% CI: 0.1280%–0.2156%). Notably, there was no difference in the frequencies of epitope-specific CD8^+^ T cells between the two epitopes in each SARS-CoV-2 vaccination group (Figure 3B). When comparing the frequencies of epitope-specific CD8^+^ T cells among the three different SARS-CoV-2 vaccine immunization groups for each epitope, the frequencies of the epitope-specific CD8^+^ T cells in individuals receiving the adenovirus-vectored vaccine seemed higher than that in the inactivated-vaccine group and the recombinant protein subunit vaccine group (Figure 3C).

### 3.5. The Frequencies of Epitope-Specific CD8^+^ T Cells Were Decreased in Individuals Receiving Two Versus One Dose of SARS-CoV-2 Vaccine

**Figure 4:**
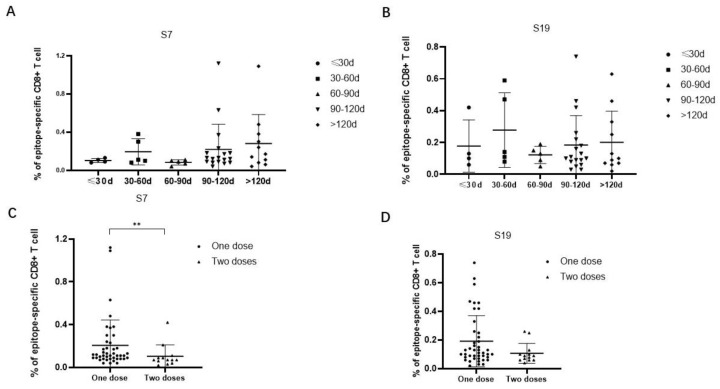
Comparison of the frequencies of epitope-specific CD8^+^ T cells between different vaccination time intervals or vaccine doses in Ad5-nCoV vaccinated individuals. (**A**,**B**) Comparison of the frequencies of epitope (**A**) S7 or (**B**) S19-specific CD8^+^ T cells at different time intervals from the latest vaccination to sample collection in Ad5-nCoV vaccinated individuals who received only one dose of adenovirus vaccine. (**C**,**D**) Comparison of the frequencies of epitope (**C**) S7 or (**D**) S19-specific CD8^+^ T cells in Ad5-nCoV vaccinated individuals received different numbers of doses of vaccine. The black lines represent the medians with the corresponding interquartile ranges. The Mann–Whitney *U* test was used for comparisons between any two groups. ** *p* < 0.01.

The frequencies of epitope-specific CD8^+^ T cells in individuals who received only one dose of the Ad5-nCoV vaccine were further analyzed according to the time interval between the latest vaccination and sample collection. The results showed that the frequencies of epitope-specific CD8^+^ T cells were elevated from a time less than 30 days to a time 30 days–60 days, then decreased at a time 60 days–90 days, and again was elevated at a time 90 days–120 days, even more than after 120 days. The trends of the frequencies of epitope-specific CD8^+^ T cells at different time intervals were similar between epitope S7 and epitope S9 (Figure 4A,B). Then, we compared the frequencies of epitope-specific CD8^+^ T cells in individuals who received different numbers of doses of the Ad5-nCoV vaccine. Notably, the frequencies of epitope-specific CD8^+^ T cells in subjects with two doses of vaccine were lower compared with that in subjects with only one dose of vaccine (*p* < 0.05 for epitope S7) (Figure 4C,D).

## 4. Discussion

SARS-CoV-2 vaccines induce specific antibody production; meanwhile, their specific antiviral T cell responses may present better protective effects for people. Therefore, exploring the vaccine-induced SARS-CoV-2-specific CD8^+^ T cell responses based on CD8^+^ T cell epitopes might be one of the important approaches to apply. In this study, we identified four nonamer epitopes on SARS-CoV-2 restricted by the HLA-E molecule that could elicit epitope-specific CD8^+^ T cell responses in a SARS-CoV-2 vaccinated population. Importantly, the HLA-E-restricted SARS-CoV-2 epitope-specific CD8^+^ T cells showed high frequencies in Ad5-nCoV vaccinated individuals. The frequencies of epitope-specific CD8^+^ T cells were decreased in individuals receiving two doses of Ad5-nCoV vaccine relative to those with only one dose. These results may greatly advance the understanding of the cellular immune defense against SARS-CoV-2 infection, and meanwhile, contribute to a more comprehensive evaluation of the protective effects of vaccines for SARS-CoV-2 covering all of the HLA-diverse populations.

The most ideal result after infection or vaccination is highly protective and lasting immunity so as to establish a high level of immunity in the population. The large-scale inoculation of populations with SARS-CoV-2 vaccines has greatly reduced the rates of infection and disease severity. The T cell response plays a key role in vaccine-mediated protection. It has been confirmed that vaccines that can induce a SARS-CoV-2-specific T cell response can effectively control infection, avoid severe tissue damage, and significantly reduce hospitalization rates and mortality. In clinical trials of a SARS-CoV-2 mRNA vaccine (BNT162b2; Modern VRC) and an adenovirus vector vaccine (AdV5; ChAdOx), SARS-CoV-2-specific CD8^+^ T cells could secrete high levels of IFN-γ, which was similar to the level of the specific CD8^+^ T cell response in patients with COVID-19. Inoculation with SARS-CoV-2 mRNA vaccine or adenovirus vector vaccine could induce a high frequency of a CD8^+^ T cell response specific to the S protein with a CCR7^-^CD45RA^+^ effect phenotype, and meanwhile, it could induce persistent memory CD8^+^ T cells [3,23,24,25]. Moreover, strong SARS-CoV-2 specific T cell responses could be detected in naturally infected individuals after vaccination. Our results showed that HLA-E restricted SARS-CoV-2 epitopes-specific CD8^+^ T cell responses could be detected in subjects vaccinated with adenovirus vector vaccine Ad5-nCoV, further indicating that Ad5-nCoV inoculation could induce effective T cell responses in the population.

The results for the detection of HLA-E-restricted SARS-CoV-2 epitope-specific CD8^+^ T cell responses showed higher frequencies in response to Ad5-nCoV than to other vaccines. We speculate that the high frequency may be related to the immunization mechanism of adenovirus vaccines. Adenoviral vector vaccines use the inherent infectivity of adenovirus to promote the expression of the S protein in vivo [26]. A portion of the S protein binds to MHC class I molecules in the cytoplasm, which can subsequently activate CD8^+^ T cells. Another part of the S protein is presented as an antigen on the surface of the cell membrane and endocytosed by other cells to combine with MHC class II molecules to activate helper T cells and B cells [27]. Conversely, the inactivated vaccine and the recombinant protein vaccine mainly induce humoral immunity. Therefore, Ad5-nCoV-vaccinated individuals showed higher frequencies of SARS-CoV-2 epitope-specific CD8^+^ T cells than individuals vaccinated with other vaccines. HLA-E has been proven to present epitopes to interact with αβTCR on CD8^+^ T cells in several infectious diseases. Since HLA-E is a ubiquitous HLA allele locus in the population, HLA-E could be used as the first choice in more specific and effective peptide vaccine design and research. The current research mainly focused on the mechanism of HLA-E-mediated NK cell function in the process of SARS-CoV-2 infection. It has been found that SARS-CoV-2 could induce HLA-E expression on the surface of pulmonary epithelial cells, which could bind peptides derived from S protein and interact with CD94/NKG2A on NK cells [28]. The peptide derived from nsp13 of SARS-CoV-2 presented by HLA-E could not interact with NKG2A on NK cells, thus mediating the killing effects of NK cells to virus-infected target cells [29]. Therefore, it is necessary to identify HLA-E-restricted epitopes of SARS-CoV-2, which would provide information for people who suffer from COVID-19 disease or have been vaccinated with SARS-CoV-2 vaccines. Based on the general rule of epitope binding motifs, nonapeptides that could be presented by HLA-E molecules always show the main anchor residues methionine (M), isoleucine (I), or leucine (L) at position two, and isoleucine (I) or leucine (L) at position nine. Therefore, the six SARS-CoV-2 nonapeptides we identified with residue M/I/L at position two and residue L at the C-terminal were conforming to the general rule of HLA-E-restricted peptides. In the context of experimental design, for the K562/HLA-E cell binding assay, we used antigen-presenting cells (K562/HLA-E cells) without peptide as a blank control and K562/HLA-E cells loaded with irrelevant peptide as a negative control to screen for the high-affinity peptide. For the IFN-γ ELISpot assay, K562/HLA-E cells without loaded peptides were used as negative controls. Notably, our study found that two epitopes S7 and S19 on SARS-CoV-2 restricted by HLA-E could also be presented by HLA-A*02, as reported in other studies [14]. Considering that 86% of the sequence was similar between the HLA-E and HLA-A*02 molecules, the HLA-A*02 molecules may share binding peptides with the HLA-E molecule. Therefore, this is a common phenomenon that HLA-E*01 and HLA-A*02 molecules could present the same epitopes, such as influenza M159-167, M158-166, and Epstein–Barr virus BZLF139-147 due to conserved deep pockets [30]. The co-presentation of antigen epitopes by both HLA-E*01 and HLA-A*02 seems to be more important in inducing CD8^+^ T cell responses for HLA-E-restricted epitopes.

It has been proven that the receptor-binding domain (RBD) on the S protein contains a variety of conformational epitopes that could induce high levels of antibody production [8], especially specific antibodies with neutralizing activity. In addition, the S protein also contains multiple dominant T cell epitopes, which could induce effective T cell immune responses [31]. Therefore, the S protein is the primary antigen used in the development of SARS-CoV-2 vaccines. The RBD region of the S protein has become the core target for research into therapeutic neutralizing antibodies and the mechanisms for specific T cell responses. The HLA-E-restricted specific CD8^+^ T cell epitopes identified in this study were all from the S protein of SARS-CoV-2. Although these epitopes aa269–aa277, aa576–aa584, aa958–aa966, and aa1185–aa1193 are not located within the RBD region (aa329–aa521), they are still important for potential applications in future.

In fact, although SARS-CoV-2 non-structural proteins (nsps) and open reading frames (ORFs) such as ORF3, nsp3, nsp4, and nsp12 show low levels in SARS-CoV-2 infected cells, they also contain very important CD8^+^ T cell epitopes. For example, ORF9b-derived epitopes could induce high-level virus-specific CD8^+^ T cell responses in COVID-19 patients, confirming good immunogenicity of these epitopes in vivo [32]. Therefore, the identification of HLA-E-restricted epitopes on nsps and ORFs of SARS-CoV-2 also makes sense for researching the effects of CD8^+^ T cells in future studies.

For the 13 individuals vaccinated with two SARS-CoV-2 vaccine doses, the HLA-E-restricted SARS-CoV-2 epitope-specific CD8^+^T cell responses were detected after both doses of vaccine. However, 6 of the 13 samples were collected less than 5 days after the second dose of the adenovirus-vectored vaccine. Since the memory T cells may not be activated shortly after immunization, this sample collection time may be quite short. Therefore, the frequencies of epitope-specific CD8^+^ T cells were lower than that after the first dose. The frequencies of epitope-specific CD8^+^ T cells after the second dose could only represent the HLA-E-restricted CD8^+^ T cell responses at a specific time point and not a long-term CD8^+^ T cell response. Furthermore, the results of the Phase 3 clinical trial of Ad5-nCoV reveal this vaccine shows good protection while producing high levels of anti-RBD antibodies and neutralizing antibodies. The levels of specific antibodies after the second dose of Ad5-nCoV vaccine may serve as evidence to demonstrate effective vaccination for Ad5-nCoV inoculated populations.

Although the HLA-E-restricted SARS-CoV-2 specific CD8^+^ T cell responses were detected in populations vaccinated with different SARS-CoV-2 vaccines, there are still some limitations of this study. In terms of subjects’ recruitment, since SARS-CoV-2 unvaccinated individuals were hard to find after the universal vaccination of the population in China, their detection in unvaccinated individuals as a background control could not be performed. The results of this study could only reflect the objective data for the HLA-E-restricted CD8^+^T cell responses among a population vaccinated with different kinds of SARS-CoV-2 vaccines at the indicated time points. Moreover, Ad5-nCoV vaccination is capable of inducing both innate and adaptive immune responses and has been demonstrated to have efficacy in protecting against symptomatic COVID-19 disease in humans. However, innate and pre-existing immunity against Ad vectors remains a serious challenge in the development and application of these vectors. In a future study of SARS-CoV-2 vaccinated and exposed populations, the controls should be set much more rigorously. Meanwhile, the vector effects induced by adenovector vaccines should be detected and considered to comprehensively evaluate the effects of these vaccines.

## 5. Conclusions

In conclusion, HLA-E-restricted SARS-CoV-2 epitope-specific CD8^+^ T cell responses could be detected in an Ad5-nCoV vaccinated population. Importantly, the levels of epitope-specific CD8^+^ T cell responses could be maintained for a long time after only one dose of vaccine. This study may be a good supplement for the evaluation of the vaccination effects of Ad5-nCoV from the perspective of T cell responses. However, HLA-E-restricted CD8^+^ T cell responses induced in naturally infected individuals after vaccination still need to be investigated in the future.

## Figures and Tables

**Table 1:** The basic information of the enrolled volunteers.

Samples ID	Gender	Age (y)	Variety of Vaccine Received	Manufacturer	Inoculation Times	Days Since Latest Inoculation
No.1	M	24	adenovirus-vectored vaccine	CanSino Biologics	1	58
No.2	M	24	adenovirus-vectored vaccine	CanSino Biologics	1	127
No.3	M	23	adenovirus-vectored vaccine	CanSino Biologics	1	54
No.4	M	31	adenovirus-vectored vaccine	CanSino Biologics	1	124
No.5	M	24	adenovirus-vectored vaccine	CanSino Biologics	1	59
No.6	M	24	adenovirus-vectored vaccine	CanSino Biologics	1	120
No.7	M	24	adenovirus-vectored vaccine	CanSino Biologics	1	128
No.8	M	24	adenovirus-vectored vaccine	CanSino Biologics	1	28
No.9	M	21	adenovirus-vectored vaccine	CanSino Biologics	1	56
No.10	M	22	adenovirus-vectored vaccine	CanSino Biologics	1	56
No.11	M	32	adenovirus-vectored vaccine	CanSino Biologics	1	27
No.12	M	22	adenovirus-vectored vaccine	CanSino Biologics	1	90
No.13	F	24	adenovirus-vectored vaccine	CanSino Biologics	1	164
No.14	M	23	adenovirus-vectored vaccine	CanSino Biologics	1	95
No.15	F	30	adenovirus-vectored vaccine	CanSino Biologics	1	159
No.16	M	24	adenovirus-vectored vaccine	CanSino Biologics	1	95
No.17	M	30	adenovirus-vectored vaccine	CanSino Biologics	1	164
No.18	M	25	adenovirus-vectored vaccine	CanSino Biologics	1	96
No.19	M	22	adenovirus-vectored vaccine	CanSino Biologics	1	91
No.20	M	32	adenovirus-vectored vaccine	CanSino Biologics	1	166
No.21	F	30	adenovirus-vectored vaccine	CanSino Biologics	1	93
No.22	M	21	adenovirus-vectored vaccine	CanSino Biologics	1	92
No.23	M	21	adenovirus-vectored vaccine	CanSino Biologics	1	92
No.24	M	22	adenovirus-vectored vaccine	CanSino Biologics	1	100
No.25	M	22	adenovirus-vectored vaccine	CanSino Biologics	1	100
No.26	M	23	adenovirus-vectored vaccine	CanSino Biologics	1	100
No.27	F	23	adenovirus-vectored vaccine	CanSino Biologics	1	165
No.28	M	21	adenovirus-vectored vaccine	CanSino Biologics	1	101
No.29	M	24	adenovirus-vectored vaccine	CanSino Biologics	1	101
No.30	M	23	adenovirus-vectored vaccine	CanSino Biologics	1	101
No.31	M	21	adenovirus-vectored vaccine	CanSino Biologics	1	101
No.32	M	24	inactivated vaccine	Sinovac Biotech	1	100
No.33	F	25	inactivated vaccine	Sinovac Biotech	1	87
No.34	F	24	inactivated vaccine	Sinovac Biotech	1	128
No.35	M	29	adenovirus-vectored vaccine	CanSino Biologics	1	171
No.36	M	26	inactivated vaccine	Sinovac Biotech	1	92
No.37	F	23	inactivated vaccine	Sinovac Biotech	1	104
No.38	M	25	inactivated vaccine	Sinovac Biotech	1	89
No.39	M	39	inactivated vaccine	China National Biotec Group Company	1	220
No.40	M	23	recombinant protein subunit vaccine	Zhifei Biological	1	27
No.41	M	24	recombinant protein subunit vaccine	Zhifei Biological	1	27
No.42	F	24	recombinant protein subunit vaccine	Zhifei Biological	1	64
No.43	F	26	recombinant protein subunit vaccine	Zhifei Biological	1	68
No.44	F	25	adenovirus-vectored vaccine	CanSino Biologics	2	3
No.45	M	32	adenovirus-vectored vaccine	CanSino Biologics	2	3
No.46	F	23	adenovirus-vectored vaccine	CanSino Biologics	2	2
No.47	F	24	adenovirus-vectored vaccine	CanSino Biologics	2	4
No.48	M	23	adenovirus-vectored vaccine	CanSino Biologics	2	4
No.49	M	23	adenovirus-vectored vaccine	CanSino Biologics	2	4
No.50	M	25	adenovirus-vectored vaccine	CanSino Biologics	2	5
No.51	M	24	adenovirus-vectored vaccine	CanSino Biologics	2	5
No.52	M	23	adenovirus-vectored vaccine	CanSino Biologics	2	5
No.53	M	24	adenovirus-vectored vaccine	CanSino Biologics	2	5
No.54	M	22	adenovirus-vectored vaccine	CanSino Biologics	2	6
No.55	M	22	adenovirus-vectored vaccine	CanSino Biologics	2	5
No.56	F	31	adenovirus-vectored vaccine	CanSino Biologics	2	6

Note: M, male; F, female. The recommended interval between the two vaccination doses of the adenovirus-vectored vaccine is 267 days.

## Data Availability

Data are contained within the article and Supplementary Materials.

## Supplementary Materials

The following supporting information can be downloaded at: https://www.mdpi.com/article/10.3390/v16010052/s1, Table S1: Information for the predicted and synthesized 36 SARS-CoV-2-derived; Table S2: Screen of SARS-CoV-2-derived HLA-E-restricted high-binding affinity peptides by the K562/HLA-E cell binding assay.

## References

[1] Li J., Lai S., Gao G.F., Shi W. (2021). The emergence, genomic diversity and global spread of SARS-CoV-2. Nature.

[2] Wu S., Zhong G., Zhang J., Shuai L., Zhang Z., Wen Z., Wang B., Zhao Z., Song X., Chen Y. (2020). A single dose of an adenovirus-vectored vaccine provides protection against SARS-CoV-2 challenge. Nat. Commun..

[3] Zhu F.-C., Li Y.-H., Guan X.-H., Hou L.-H., Wang W.-J., Li J.-X., Wu S.-P., Wang B.-S., Wang Z., Wang L. (2020). Safety, tolerability, and immunogenicity of a recombinant adenovirus type-5 vectored COVID-19 vaccine: A dose-escalation, open-label, non-randomised, first-in-human trial. Lancet.

[4] Chi X., Guo Y., Zhang G., Sun H., Zhang J., Li M., Chen Z., Han J., Zhang Y., Zhang X. (2022). Broadly neutralizing antibodies against Omicron-included SARS-CoV-2 variants induced by vaccination. Signal Transduct. Target. Ther..

[5] Zhu F.-C., Guan X.-H., Li Y.-H., Huang J.-Y., Jiang T., Hou L.-H., Li J.-X., Yang B.-F., Wang L., Wang W.-J. (2020). Immunogenicity and safety of a recombinant adenovirus type-5-vectored COVID-19 vaccine in healthy adults aged 18 years or older: A randomised, double-blind, placebo-controlled, phase 2 trial. Lancet.

[6] Li J., Hou L., Guo X., Jin P., Wu S., Zhu J., Pan H., Wang X., Song Z., Wan J. (2022). Heterologous AD5-nCOV plus CoronaVac versus homologous CoronaVac vaccination: A randomized phase 4 trial. Nat. Med..

[7] Wu S., Huang J., Zhang Z., Wu J., Zhang J., Hu H., Zhu T., Zhang J., Luo L., Fan P. (2021). Safety, tolerability, and immunogenicity of an aerosolised adenovirus type-5 vector-based COVID-19 vaccine (Ad5-nCoV) in adults: Preliminary report of an open-label and randomised phase 1 clinical trial. Lancet Infect. Dis..

[8] Terreri S., Mortari E.P., Vinci M.R., Russo C., Alteri C., Albano C., Colavita F., Gramigna G., Agrati C., Linardos G. (2022). Persistent B cell memory after SARS-CoV-2 vaccination is functional during breakthrough infections. Cell Host Microbe.

[9] Edridge A.W.D., Kaczorowska J., Hoste A.C.R., Bakker M., Klein M., Loens K., Jebbink M.F., Matser A., Kinsella C.M., Rueda P. (2020). Seasonal coronavirus protective immunity is short-lasting. Nat. Med..

[10] Braun J., Loyal L., Frentsch M., Wendisch D., Georg P., Kurth F., Hippenstiel S., Dingeldey M., Kruse B., Fauchere F. (2020). SARS-CoV-2-reactive T cells in healthy donors and patients with COVID-19. Nature.

[11] Schulien I., Kemming J., Oberhardt V., Wild K., Seidel L.M., Killmer S., Sagar N., Daul F., Salvat Lago M., Decker A. (2021). Characterization of pre-existing and induced SARS-CoV-2-specific CD8(+) T cells. Nat. Med..

[12] Thevarajan I., Nguyen T.H.O., Koutsakos M., Druce J., Caly L., van de Sandt C.E., Jia X., Nicholson S., Catton M., Cowie B. (2020). Breadth of concomitant immune responses prior to patient recovery: A case report of non-severe COVID-19. Nat. Med..

[13] McMahan K., Yu J., Mercado N.B., Loos C., Tostanoski L.H., Chandrashekar A., Liu J., Peter L., Atyeo C., Zhu A. (2021). Correlates of protection against SARS-CoV-2 in rhesus macaques. Nature.

[14] Habel J.R., Nguyen T.H., van de Sandt C.E., Juno J.A., Chaurasia P., Wragg K., Koutsakos M., Hensen L., Jia X., Chua B. (2020). Suboptimal SARS-CoV-2-specific CD8(+) T cell response associated with the prominent HLA-A*02:01 phenotype. Proc. Natl. Acad. Sci. USA.

[15] Sekine T., Perez-Potti A., Rivera-Ballesteros O., Strålin K., Gorin J.-B., Olsson A., Llewellyn-Lacey S., Kamal H., Bogdanovic G., Muschiol S. (2020). Robust T Cell Immunity in Convalescent Individuals with Asymptomatic or Mild COVID-19. Cell.

[16] Peng Y., Felce S.L., Dong D., Penkava F., Mentzer A.J., Yao X., Liu G., Yin Z., Chen J.-L., Lu Y. (2022). An immunodominant NP105-113-B*07:02 cytotoxic T cell response controls viral replication and is associated with less severe COVID-19 disease. Nat. Immunol..

[17] Joosten S.A., Sullivan L.C., Ottenhoff T.H. (2016). Characteristics of HLA-E Restricted T-Cell Responses and Their Role in Infectious Diseases. J. Immunol. Res..

[18] Bansal A., Gehre M.N., Qin K., Sterrett S., Ali A., Dang Y., Abraham S., Costanzo M.C., Venegas L.A., Tang J. (2021). HLA-E-restricted HIV-1-specific CD8+ T cell responses in natural infection. J. Clin. Investig..

[19] Sharpe H.R., Bowyer G., Brackenridge S., Lambe T. (2019). HLA-E: Exploiting pathogen-host interactions for vaccine development. Clin. Exp. Immunol..

[20] Kraemer T., Blasczyk R., Bade-Doeding C. (2014). HLA-E: A novel player for histocompatibility. J. Immunol. Res..

[21] Caccamo N., Sullivan L.C., Brooks A.G., Dieli F. (2020). Harnessing HLA-E-restricted CD8 T lymphocytes for adoptive cell therapy of patients with severe COVID-19. Br. J. Haematol..

[22] Yuling W., Boquan J., Na L., Ying T., Yun D., Qi L., Ying M., Ran Z. (2023). Preparation and identification of monoclonal antibodies against structural proteins of SARS-CoV-2. J. Air Force Med. Univ..

[23] Sahin U., Muik A., Vogler I., Derhovanessian E., Kranz L.M., Vormehr M., Quandt J., Bidmon N., Ulges A., Baum A. (2021). BNT162b2 vaccine induces neutralizing antibodies and poly-specific T cells in humans. Nature.

[24] Reynolds C.J., Pade C., Gibbons J.M., Otter A.D., Lin K.M., Muñoz Sandoval D., Pieper F.P., Butler D.K., Liu S., Joy G. (2022). Immune boosting by B.1.1.529 (Omicron) depends on previous SARS-CoV-2 exposure. Science.

[25] Rodda L.B., Morawski P.A., Pruner K.B., Fahning M.L., Howard C.A., Franko N., Logue J., Eggenberger J., Stokes C., Golez I. (2022). Imprinted SARS-CoV-2-specific memory lymphocytes define hybrid immunity. Cell.

[26] Jacob-Dolan C., Barouch D.H. (2022). COVID-19 Vaccines: Adenoviral Vectors. Annu. Rev. Med..

[27] Sakurai F., Tachibana M., Mizuguchi H. (2022). Adenovirus vector-based vaccine for infectious diseases. Drug Metab. Pharmacokinet..

[28] Bortolotti D., Gentili V., Rizzo S., Rotola A., Rizzo R. (2020). SARS-CoV-2 Spike 1 Protein Controls Natural Killer Cell Activation via the HLA-E/NKG2A Pathway. Cells.

[29] Hammer Q., Dunst J., Christ W., Picarazzi F., Wendorff M., Momayyezi P., Huhn O., Netskar H.K., Maleki K.T., García M. (2022). SARS-CoV-2 Nsp13 encodes for an HLA-E-stabilizing peptide that abrogates inhibition of NKG2A-expressing NK cells. Cell Rep..

[30] Grant E.J., Nguyen A.T., Lobos C.A., Szeto C., Chatzileontiadou D.S., Gras S. (2020). The unconventional role of HLA-E: The road less traveled. Mol. Immunol..

[31] Tennøe S., Gheorghe M., Stratford R., Clancy T. (2022). The T Cell Epitope Landscape of SARS-CoV-2 Variants of Concern. Vaccines.

[32] Weingarten-Gabbay S., Klaeger S., Sarkizova S., Pearlman L.R., Chen D.Y., Gallagher K.M., Bauer M.R., Taylor H.B., Dunn W.A., Tarr C. (2021). Profiling SARS-CoV-2 HLA-I peptidome reveals T cell epitopes from out-of-frame ORFs. Cell.

