# Supplementary material for: Ad5-nCoV Vaccination Could Induce HLA-E Restricted CD8+ T Cell Responses Specific for Epitopes on Severe Acute Respiratory Syndrome Coronavirus 2 Spike Protein
Source: Viruses. 2023 Dec 28;16(1):52. doi: 10.3390/v16010052 (PMC10820189; doi:10.3390/v16010052)
Supplement: Supplementary file 1 [file viruses-16-00052-s001.zip › viruses-2735181-supplementary.pdf]

Table S1. Information for the predicted and synthesized 36 SARS-CoV-2-derived HLA-E-restricted nonapeptides

NO.	Protein	Start	End	Peptide sequence	IEDB score	NetMHCpan %Rank Bind Level	NetMHC %Rank Bind Level
					HLA-E*0101	HLA-E*0101 and E*0103	HLA-E*0101
1	S1	78	86	RFDNPVLPF	0.121202	0.478 <= SB	-
2	S2	109	117	TLDSKTQSL	0.271885	0.022 <= SB	0.70 <= WB
3	S3	171	179	VSQPFLMDL	0.083054	0.399 <= SB	1.40 <= WB
4	S4	202	210	KIYSKHTPI	0.07487	0.293 <= SB	-
5	S5	204	212	YSKHTPINL	0.095193	0.104 <= SB	-
6	S6	233	241	INITRFQTL	0.080026	0.117 <= SB	1.40 <= WB
7	S7	269	277	YLQPRTFLL	0.545607	0.009 <= SB	0.01 <= SB
8	S8	327	335	VRFPNITNL	0.096126	0.254 <= SB	1.20 <= WB
9	S9	382	390	VSPTKLNDL	0.102509	0.176 <= SB	1.30 <= WB
10	S10	417	425	KIADYNYKL	0.234359	0.233 <= SB	-
11	S11	505	513	YQPYRVVVL	0.290624	0.009 <= SB	0.03 <= SB
12	S12	525	533	CGPKKSTNL	0.035989	0.245 <= SB	1.80 <= WB
13	S13	576	584	VRDPQTLEI	0.067788	0.238 <= SB	1.10 <= WB
14	S14	584	592	ILDITPCSF	0.150685	0.370 <= SB	-
15	S15	786	794	KQIYKTPPI	0.044301	0.327 <= SB	1.00 <= WB

16	S16	894	902	LQIPFAMQM	0.061265	0.443 <= SB	0.60 <= WB
17	S17	915	923	VLYENQKLI	0.095694	0.359 <= SB	-
18	S18	951	959	VVNQNAQAL	0.132208	0.423 <= SB	1.80 <= WB
19	S19	958	966	ALNTLVKQL	0.060342	0.414 <= SB	-
20	S20	976	984	VLNDILSRL	0.155449	0.222 <= SB	-
21	S21	1004	1012	LQTYVTQQL	0.02558	0.467 <= SB	1.90 <= WB
22	S22	1054	1062	QSAPHGVVF	0.110504	0.179 <= SB	1.10 <= WB
23	S23	1055	1063	SAPHGVVFL	0.220321	0.052 <= SB	1.20 <= WB
24	S24	1137	1145	VYDPLQPEL	0.22485	0.021 <= SB	0.30 <= SB
25	S25	1185	1193	RLNEVAKNL	0.147363	0.194 <= SB	-
26	N1	159	167	LQLPQGTTL	0.095175	0.077 <= SB	0.25 <= SB
27	N2	266	274	KAYNVTQAF	0.105461	0.336 <= SB	-
28	N3	307	315	FAPSASAFF	0.122449	0.393 <= SB	-
29	N4	338	346	KLDDKDPNF	0.254909	0.117 <= SB	-
30	M1	101	109	RLFARTRSM	0.21133	0.140 <= SB	0.70 <= WB
31	M2	111	119	SFNPETNIL	0.100966	0.394 <= SB	1.10 <= WB
32	M3	112	120	FNPETNILL	0.060525	0.090 <= SB	0.50 <= SB
33	M4	121	129	NVPLHGTIL	0.096825	0.472 <= SB	0.90 <= WB
34	M5	212	220	SSSDNIALL	0.172655	0.070 <= SB	-
35	M6	213	221	SSDNIALLV	0.067993	0.408 <= SB	-
36	E1	57	65	YVYSRVKNL	0.206633	0.074 <= SB	-

Note: WB, a weak binding; SB, a strong binding.

Table S2. Screen of SARS-CoV-2 -derived HLA-E-restricted high-binding affinity peptides by the K562/HLA-E cell binding assay

NO. peptide	Protein	Location	Sequence	IEDB	NetMHC	NetMHCpan	K562/HLA-E*0103 binding assay	K562/HLA-E*0101 binding assay
				HLA-E*0101	HLA-E*0101	HLA-E*0101 and E*0103	FI	FI
S7	S protein	aa269-aa277	YLQPRTFLL	0.545607	0.009 <= SB	0.01 <= SB	1.022	1.871
S10	S protein	aa417-aa425	KIADYNYKL	0.234359	0.233 <= SB	-	-	1.254
S13	S protein	aa576-aa584	VRDPQTLEI	0.067788	0.238 <= SB	1.10 <= WB	1.160	1.122
S15	S protein	aa786-aa794	KQIYKTPPI	0.044301	0.327 <= SB	1.00 <= WB	-	1.644
S19	S protein	aa958-aa966	ALNTLVKQL	0.060342	0.414 <= SB	-	1.508	1.051
S25	S protein	aa1185-aa1193	RLNEVAKNL	0.147363	0.194 <= SB	-	1.209	1.124

HLA-A2 leading sequences (positive control)	-	-	VMAPRTLIL/VMAPRTLVL	-	-	-	2.977	3.245
MAGE-1 (negative control)	-	-	EADPTGHSY	-	-	-	0.174	0.340

Note: WB, a weak binding; SB, a strong binding. MAGE-1, Melanoma-associated antigen 1. FI, fluorescence index, which was determined as follows: FI = (mean PE fluorescence with the given peptide - mean PE fluorescence without peptide)/ (mean PE fluorescence without peptide). FI ≥ 1 represents high-affinity peptides.
